# Bodenhamer on Cholera

**Published:** 1855-09

**Authors:** W. Bodenhamer


### BODENHAMER ON CHOLERA.

The following article in relation to the Epidemic Cholera, as it appeared in New Orleans in the winter of 1848, is from the pen of Dr. Bodenhamer, now of this city. It is copied from the Louisville Democrat of June 12th, 1849. Dr. Bodenhamer has retired from the general practice of medicine altogether, and is now consulting surgeon for fistulous affections and kindred ailments, therefore what he says may well be regarded as the unbiassed observations of a lover of truth.

Gentlemen:—As our city may sooner or later be visited by the malignant epidemic which is evidently on our borders, permit me to make a few practical remarks relative to its causes and its prevention. It is not my intention to enter into any protracted scientific details or analysis, but merely submit such reflections as have occurred to my mind since my return from New Orleans, where this dread scourge prevailed in a most fatal form for some time previous to my departure.

The local predisposing causes of malignant cholera are so well known, and have of late been so ably discussed here, that it is scarcely necessary to repeat them. A marked combination of these was eminently conspicuous in that unfortunate and afflicted city, inducing the "*epidemic constitution*," and tending greatly to develop the disease there, both in numbers and in intensity. For some time previous to the outbreak of the malady, and during its early prevalence, the mercury ranged at summer heat, varying sometimes within the twenty-four hours a few degrees above and below that temperature. There was most of the time a drizzling, soaking rain, which completely saturated the earth, kept the streets in a most miserable condition, and drenched all who were exposed in the open air. Indeed there was a diversity of climate, of season, and of other causes, during the time, sufficient almost for the production of any disease. The streets, the alleys, the yards, the levee, the lots, &c., were in a most shocking filthy condition, filling the atmosphere with a poisonous exhalation unsurpassed, perhaps, by the banks of the Ganges; so much so that the effluvia and the emanations of the city became a subject of general complaint and of universal notoriety. Now, it is an admitted fact, that nothing so powerfully predisposes to malignant cholera as the combination of humidity with impurity of the atmosphere; humid and contaminated air being the medium in which the poison chiefly lurks and propagates; hence, in no other city, perhaps, in the United States, was the disease ever so fatal in proportion to the number attacked, and perhaps no other city in our country was ever so notoriously deficient in hygienic precautions and in sanitary measures.

The appalling mortality, however, at the Charity Hospital, was obviously owing to the exposed state of the patients previous to admission, and to the fact that none but the worst cases, from among the poorest and most abandoned or degraded of the population, were taken there. They were persons of exposed life, of intemperate habits, and who subsisted on miserable or raw kinds of food, and who entirely neglected the premonitory stage of the disease. There being no other institution in that large city in which the indigent could be admitted, many of these poor and unfortunate creatures had to be conveyed in open vehicles for upwards of a mile, under the influence of alarm and exposed to the inclemencies of the weather, and to the jolting motions of a cart or dray; all of which are well calculated to increase the diarrhœa and precipitate the state of collapse: hence many cases were admitted some hours after the disease had been fully developed, the pulse being imperceptible, or nearly so, the skin cold, damp and livid, and the features sunken.

The deplorable state of things that existed at New Orleans, on the advent of the epidemic there, should admonish us to guard against, and to avoid as much as possible, the same causes. We, as well as our municipal authorities, should remember the important fact, that this mortal disease acquires magnitude and strength in proportion to our ignorance and neglect of its causes and prevention. It is true the constituted authorities of our city cannot bar-out cholera by any quarantine regulations, however rigidly enforced; they cannot limit it by cordons and by baricades, but they can do very much to weaken its force, if not prevent it altogether, by adopting and rigidly enforcing good and substantial sanitary measures. They can give us uncontaminated air and pure water, by giving us clean streets, clean alleys, clean yards, clean sewers, &c. They, however, already deserve the highest commendation for their valuable and praiseworthy efforts towards the accomplishment of this all important object; the beneficial results of which, I am well convinced, will be very evident, very apparent, whether the epidemic visits us or not. They only need, for the completion of their excellent regulations, an efficient plan for checking the ravages of the disease amongst the poor and unfortunate—for the rich will take care of themselves—and that is, the establishment, at the first outbreak of cholera, of temporory ward hospitals or receiving houses, with properly qualified physicians to attend to them. These houses should be sufficiently numerous, and at such distances as to allow as little time as possible to be lost by those who are attacked.

As it is now a settled point that cholera is not a contagious disease in the strict sense of that term, and as its causes and its propagation are now rendered so much more definite and uniform, having been subjected to the keenest discussion—and as its treatment, likewise, is now so much better understood by the profession—what good reason can be assigned, then, for the great excitement and the great alarm which so usually prevail wherever the epidemic appears? It must be evidently owing in part to the rapid and fatal effects of the disease, but doubtless much more so to the unmanly fear of contagion, which always brings alarm and terror in its train, producing a panic or species of "*choleraphobia*," which is scarcely less to be dreaded than cholera itself, being a powerful predisposing cause. Let no person then be frightened into cholera by unnecessary excitement, fear or alarm, but let each one have a firm and an abiding belief that it is entirely in his own power to prevent an attack, by attention to diet, to warm clothing, to cleanliness of person, to the use of baths and other means of ablution, to moderation in exercise, both mental and corporeal, to the ventilation of sleeping apartments, to the avoidance of undue exposure in wet or damp clothes; and, in short, to everything that conduces to tranquility of mind and to soundness of body. By observing these strictly, with a hopeful disposition and a peaceful and cheerful conscience, a person may with propriety consider himself comparatively secure from an attack. All should reflect seriously how much easier it is to prevent than to cure cholera. The idea, however, of preventing an attack by taking any medicine whatever, when feeling perfectly well, is too absurd for a moment's consideration. Nothing is better calculated to invite an attack than such a pernicious practice. In what better condition could a person be to resist disease than in perfect health? But during *choleraphobia* many persons are not satisfied with this; they must needs be teazing their stomachs continually with some *villainous compound* or other to make themselves better than well. All such should remember the significant epitaph inscribed (at his own request) upon the tomb of the Italian Count, who, like themselves, experimented with his health under the apprehension of disease:

> "I was well;
> Would be better;
> Took physic,
> And died."

The best preventive is to pursue a temperate course in all things, shunning all cholera medicines as preventives while in health. As soon, however, as any of the symptoms of the disease appear, then, and not till then, should any medicine be taken. The patient should at once send for his own family physician or for some one in whose skill and knowledge he has full confidence, for faith and confidence are powerful adjuncts in overcoming disease. The advice of such a physician should also previously be obtained with regard to the domestic treatment of this malady in its earliest stage, in case of sudden attack and while waiting for him. The premonitory diarrhœa, almost invariably a precursor of the disease, may, however, often at once be arrested by remaining at home, maintaining the recumbent or horizontal posture, living on crackers and tea and taking some very mild medicine. Nothing will so soon check this diarrhœa, or any other, as confinement to bed, external warmth, perfect quietude and *starvation*, with some of our common opiates, cordials or astringents. The constant attendance of a physician is, however, indispensably necessary in all the stages of this fearful, rapid, and too often fatal disease; for to counteract it, the treatment must be prompt, the medicine must be quick, powerful and permanent in its effect. It must be quick, lest the disease shall have progressed beyond medical jurisdiction; it must be powerful, lest it be carried before the progress of the disease as a straw in the current of the ocean; and it must be permanent, lest the disease, after a short and deceptive remission, return with greater violence than at first.

It is injurious to make any great change in the ordinary mode of living, with the exception of fruit and vegetables; and reason and experience dictate the propriety of refraining from these altogether, or to be very sparing indeed in their use. Vinous, malt or fermented liquors should be avoided, but the very best quality of brandy, rum, gin, or "*old Bourbon*" might, in moderation, be used. It is, however, very far from my wish, by this, to recommend intemperance, either directly or indirectly; but I do not hesitate to state that the occasional use of such stimuli might be found highly serviceable during the prevalence of this mortal pestilence. I need hardly remark, that the habitually intemperate lose all the benefits of this remedy. The use of large quantities of fluids during the epidemic is also objectionable; therefore, persons should be careful "not to take too much water in their brandy." No purgative medicines whatever, (especially the saline) unless absolutely necessary, should be used for the purpose simply of obviating constipation of the bowels. This condition, if possible, should be obviated simply by diet and by injections. Persons should also shun as they would the pestilence itself, all cholera nostrums of which they neither know the nature nor the composition; and should physicians recommend such articles to their patients without any knowledge, they would thereby indirectly admit that they themselves had no confidence in their own prescriptions, and that they were ignorant of the healing art.

With many apologies for having occupied so much space, I have the honor to be,

Gentlemen, your obedient servant,

W. BODENHAMER, M. D.

---

As our Journal is taken in some families who favor Homœopathy, we publish for their benefit the following article entire. It certainly contains sound advice, and may be safely and profitably followed by all, provided every effort is made to ensure the earliest attention of a physician:

HOMŒOPATHIC INSTRUCTIONS FOR FAMILIES WITH REFERENCE TO THE CHOLERA.

At a meeting of the Hahnemann Academy of Medicine, held July 16, 1854, the Committee on Cholera reported the following instructions for the domestic management of this disease:

1. Avoid crowded assemblies and crowded sleeping apartments, and as much as possible shun the presence of filthy persons, for the disease is mostly developed in crowded dwellings, ships, prisons, camps, &c.

2. Observe cleanliness of person and enjoin the same upon your household.

3. Dwellings—especially the sleeping apartments—should in all cases be thoroughly ventilated.

4. Pursue your ordinary course of diet, observing some moderation as to vegetables and fruits. Night meals are to be avoided. Regularity in the hours of eating is very desirable. Alcoholic drinks are objectionable, the intemperate being particularly liable to this disease. Ice-water and ices should be used with extreme moderation. Articles of diet known to disagree with the regular action of the bowels should be most scrupulously avoided.

5. Avoid mental or bodily excitement or fatigue. Keep the person warmly clad.

6. Cathartics and laxatives must be wholly avoided. No means should be taken to remove constipation, except such as are prescribed by a physician. The use of laudanum, opium, or cholera mixtures of any kind is hazardous.

7. It is better to take no medicine as preventative of cholera, but the slightest derangement of the bowels should be met by appropriate treatment.

8. Should there be oppression or sickness at the stomach, shiverings or dizziness, with or without relaxed bowels, *Ipecac* of the second or third trituration or dilution, may be taken every two or three hours.

9. If there be watery looseness of the bowels, with or without nausea, pain or cramps, take one drop of *Veratrum*, first dilution, every half hour or hour.

10. If the diarrhœa should become profuse, with or without pain or vomiting, discharges very frequent, being watery or resembling rice-water, with or without cramps, coldness, and blueness, with rapid sinking, take one or two drops of the spirits of camphor every five or ten minutes until reaction takes place.

From the moment the diarrhœa becomes urgent, the patient should go to bed and be well wrapped with blankets. Bottles of hot water should be applied to the feet, and medical aid at once be summoned. No external use of camphor is advisable while other remedies are employed.

Published by order of the Hahnemann Academy of Medicine, New York, July 17, 1854.

